# Addiction is a Reward Deficit and Stress Surfeit Disorder

**DOI:** 10.3389/fpsyt.2013.00072

**Published:** 2013-08-01

**Authors:** George F. Koob

**Affiliations:** ^1^Committee on the Neurobiology of Addictive Disorders, The Scripps Research Institute, La Jolla, CA, USA

**Keywords:** opponent process, extended amygdala, corticotropin-releasing factor, dynorphin, reward, compulsive, withdrawal, prefrontal cortex

## Abstract

Drug addiction can be defined by a three-stage cycle – *binge/intoxication*, *withdrawal/negative affect*, and *preoccupation/anticipation* – that involves allostatic changes in the brain reward and stress systems. Two primary sources of reinforcement, positive and negative reinforcement, have been hypothesized to play a role in this allostatic process. The negative emotional state that drives negative reinforcement is hypothesized to derive from dysregulation of key neurochemical elements involved in the brain reward and stress systems. Specific neurochemical elements in these structures include not only decreases in reward system function (within-system opponent processes) but also recruitment of the brain stress systems mediated by corticotropin-releasing factor (CRF) and dynorphin-κ opioid systems in the ventral striatum, extended amygdala, and frontal cortex (both between-system opponent processes). CRF antagonists block anxiety-like responses associated with withdrawal, block increases in reward thresholds produced by withdrawal from drugs of abuse, and block compulsive-like drug taking during extended access. Excessive drug taking also engages the activation of CRF in the medial prefrontal cortex, paralleled by deficits in executive function that may facilitate the transition to compulsive-like responding. Neuropeptide Y, a powerful anti-stress neurotransmitter, has a profile of action on compulsive-like responding for ethanol similar to a CRF_1_ antagonist. Blockade of the κ opioid system can also block dysphoric-like effects associated with withdrawal from drugs of abuse and block the development of compulsive-like responding during extended access to drugs of abuse, suggesting another powerful brain stress system that contributes to compulsive drug seeking. The loss of reward function and recruitment of brain systems provide a powerful neurochemical basis that drives the compulsivity of addiction.

## What is Addiction?

Addiction can be defined as a chronic, relapsing disorder that has been characterized by (i) a compulsion to seek and take drugs, (ii) loss of control over drug intake, and (iii) emergence of a negative emotional state (e.g., dysphoria, anxiety, and irritability) that defines a motivational withdrawal syndrome when access to the drug is prevented (1). The occasional, limited, recreational use of a drug is clinically distinct from escalated drug use, the loss of control over drug intake, and the emergence of compulsive drug-seeking behavior that characterize addiction.

**Figure 1:**
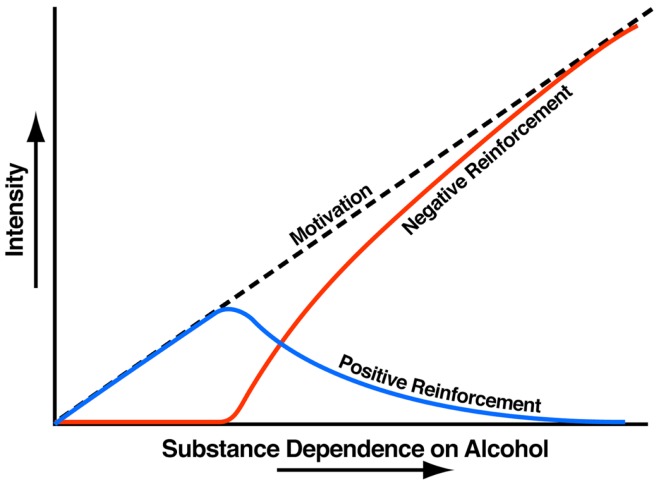
**Theoretical framework relating addiction cycle to motivation for drug seeking**. The figure shows the change in the relative contribution of positive and negative reinforcement constructs during the development of substance dependence [taken with permission from Ref. (61)].

Addiction has been conceptualized as a three-stage cycle – *binge/intoxication*, *withdrawal/negative affect*, and *preoccupation/anticipation* – that worsens over time and involves allostatic changes in the brain reward and stress systems. Two primary sources of reinforcement, positive and negative reinforcement, have been hypothesized to play a role in this allostatic process. Positive reinforcement is defined as the process by which presentation of a stimulus increases the probability of a response; negative reinforcement is defined as the process by which removal of an aversive stimulus (or negative emotional state of withdrawal in the case of addiction) increases the probability of a response. Reward is operationally defined similarly to positive reinforcement as any stimulus that increases the probability of a response but also has a positive hedonic effect. Different theoretical perspectives from experimental psychology (positive and negative reinforcement frameworks), social psychology (self-regulation failure framework), and neurobiology (counteradaptation and sensitization frameworks) can be superimposed on the stages of the addiction cycle (1). These stages are thought to feed into each other, become more intense, and ultimately lead to the pathological state known as *addiction* (Figure 1). The neural substrates for the two sources of reinforcement that play a key role in the allostatic neuroadaptations derive from two key motivational systems required for survival: the brain reward and brain stress systems.

## Brain Reward Systems

**Figure 2:**
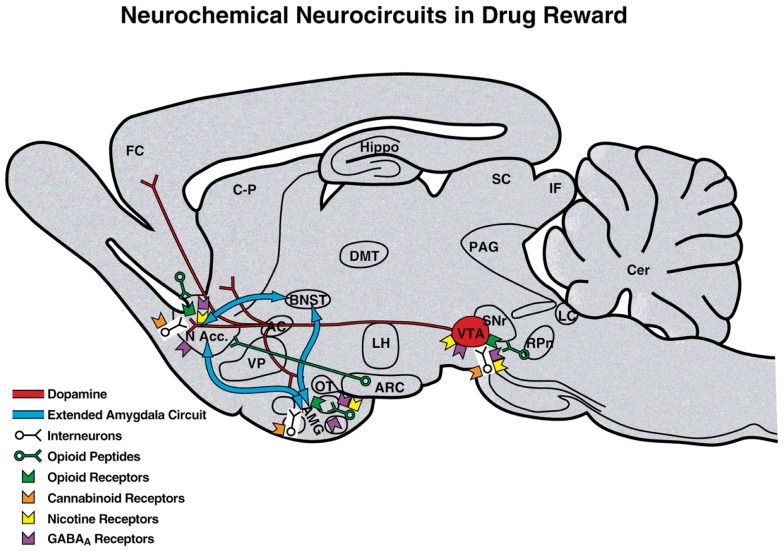
**Neurotransmitter pathways and receptor systems implicated in the acute reinforcing effects of drugs of abuse within the medial forebrain bundle**. A sagittal rodent brain section is shown. The medial forebrain bundle represents ascending and descending projections between the ventral forebrain (nucleus accumbens, olfactory tubercle, and septal area) and ventral midbrain (ventral tegmental area; not shown in figure for clarity). Cocaine and amphetamines increase dopamine levels in the nucleus accumbens and amygdala via direct actions on dopamine terminals. Opioids activate endogenous opioid receptors in the ventral tegmental area, nucleus accumbens, and amygdala. Opioids also facilitate the release of dopamine in the nucleus accumbens via actions either in the ventral tegmental area or nucleus accumbens but are also hypothesized to activate elements independent of the dopamine system. Alcohol activates GABA_A_ receptors or enhances GABA release in the ventral tegmental area, nucleus accumbens, and amygdala. Alcohol is also hypothesized to facilitate the release of opioid peptides in the ventral tegmental area, nucleus accumbens, and central nucleus of the amygdala. Alcohol facilitates the release of dopamine in the nucleus accumbens via an action either in the ventral tegmental area or nucleus accumbens. Nicotine activates nicotinic acetylcholine receptors in the ventral tegmental area, nucleus accumbens, and amygdala either directly or indirectly via actions on interneurons. Cannabinoids activate cannabinoid CB_1_ receptors in the ventral tegmental area, nucleus accumbens, and amygdala. Cannabinoids facilitate the release of dopamine in the nucleus accumbens via an unknown mechanism, either in the ventral tegmental area or nucleus accumbens. The blue arrows represent the interactions within the extended amygdala system hypothesized to play a key role in psychostimulant reinforcement. AC, anterior commissure; AMG, amygdala; ARC, arcuate nucleus; BNST, bed nucleus of the stria terminalis; Cer, cerebellum; C-P, caudate-putamen; DMT, dorsomedial thalamus; FC, frontal cortex; Hippo, hippocampus; IF, inferior colliculus; LC, locus coeruleus; LH, lateral hypothalamus; MFB, medial forebrain bundle; N Acc., nucleus accumbens; OT, olfactory tract; PAG, periaqueductal gray; RPn, reticular pontine nucleus; SC, superior colliculus; SNr, substantia nigra pars reticulata; VP, ventral pallidum; VTA, ventral tegmental area [taken with permission from Ref. (183)].

Comprehension of a brain reward system was greatly facilitated by the discovery of electrical brain stimulation reward by Olds and Milner (2). Brain stimulation reward involves widespread neurocircuitry throughout the brain, but the most sensitive sites include the trajectory of the medial forebrain bundle that connects the ventral tegmental area with the basal forebrain [(2–4); Figure 2]. All drugs of abuse acutely decrease brain stimulation reward thresholds [i.e., increase or facilitate reward; (5)]. When drugs are administered chronically, withdrawal from drugs of abuse increases reward thresholds (decrease reward). Although much emphasis was initially placed on the role of ascending monoamine systems, particularly the dopamine system, in the medial forebrain bundle in mediating brain stimulation reward, other non-dopaminergic systems in the medial forebrain bundle clearly play a key role (6–8). Indeed, the role of dopamine is hypothesized to be more indirect. Many studies suggest that activation of the mesolimbic dopamine system attaches incentive salience to stimuli in the environment (9–11) to drive the performance of goal-directed behavior (12) or activation in general (13, 14), and work concerning the acute reinforcing effects of drugs of abuse supports this hypothesis.

Our knowledge of the neurochemical substrates that mediate the acute reinforcing effects of drugs of abuse has contributed significantly to our knowledge of the brain reward system. These substrates include connections of the medial forebrain bundle reward system with primary contributions from the ventral tegmental area, nucleus accumbens, and amygdala. Much evidence supports the hypothesis that psychostimulant drugs dramatically activate the mesolimbic dopamine system (projections from the ventral tegmental area to the nucleus accumbens) during limited-access drug self-administration and that this mechanism is critical for mediating the rewarding effects of cocaine, amphetamines, and nicotine. However, evidence supports both dopamine-dependent and dopamine-independent neural substrates for opioid and alcohol reward (15–17). Serotonin systems, particularly those involving serotonin 5-HT_1B_ receptor activation in the nucleus accumbens, have also been implicated in the acute reinforcing effects of psychostimulant drugs, whereas μ-opioid receptors in both the nucleus accumbens and ventral tegmental area mediate the reinforcing effects of opioids. Opioid peptides in the ventral striatum and amygdala have been hypothesized to mediate the acute reinforcing effects of ethanol self-administration, largely based on the effects of opioid antagonists. Inhibitory γ-aminobutyric acid (GABA) systems are activated both pre- and postsynaptically in the amygdala by ethanol at intoxicating doses, and GABA receptor antagonists block ethanol self-administration [for comprehensive reviews, see (16, 17)].

For the *binge/intoxication stage* of the addiction cycle, studies of the acute reinforcing effects of drugs of abuse *per se* have identified key neurobiological substrates. Evidence is strong for a role for dopamine in the acute reinforcing actions of psychostimulants, opioid peptide receptors in the acute reinforcing effects of opioids, and GABA and opioid peptides in the acute reinforcing actions of alcohol. Important anatomical circuits include the mesocorticolimbic dopamine system that originates in the ventral tegmental area and local opioid peptide systems, both of which converge on the nucleus accumbens (17).

## Brain Stress Systems

The brain stress systems can be defined as neurochemical systems that are activated during exposure to acute stressors or in a chronic state of stress and mediate species-typical behavioral responses. These behavioral responses in animals range from freezing to flight and typically have face and predictive validity for similar behavior responses in humans. For example, animals exposed to a stressor will show an enhanced freezing response to a conditioned fear stimulus, an enhanced startle response to a startle stimulus, avoidance of open areas, open arms, or height, and enhanced species-typical responses to an aversive stimulus (e.g., burying a shock probe in the defensive burying test). Key neuronal/neurochemical systems with circumscribed neurocircuitry that mediate behavioral responses to stressors include glucocorticoids, corticotropin-releasing factor (CRF), norepinephrine, and dynorphin, and key neurochemical systems that act in opposition to the brain stress systems include neuropeptide Y (NPY), nociceptin, and endocannabinoids [for reviews, see (18–20)]. For the purposes of this review, two brain stress systems with prominent roles in driving the dark side of addiction will be considered: CRF and dynorphin.

### Corticotropin-releasing factor

Corticotropin-releasing factor is a 41-amino-acid polypeptide that controls hormonal, sympathetic, and behavioral responses to stressors (21, 22). Central administration of CRF mimics the behavioral response to activation and stress in rodents, and administration of competitive CRF receptor antagonists generally has anti-stress effects [for reviews, see (23–26)]. Two major CRF receptors have been identified, with CRF_1_ receptor activation associated with increased stress responsiveness (27) and CRF_2_ receptor activation associated with decreases in feeding and decreases in stress responsiveness (28, 29), although there is some controversy in this area (30). CRF neurons are present in the neocortex, the extended amygdala, the medial septum, the hypothalamus, the thalamus, the cerebellum, and autonomic midbrain and hindbrain nuclei (31). Extensive research has been performed on CRF neurons in the paraventricular nucleus of the hypothalamus (PVN), central nucleus of the amygdala (CeA), and bed nucleus of the stria terminalis (BNST), demonstrating a key role for PVN CRF neurons in controlling the pituitary adrenal response to stress (32) and a key role for BNST and CeA CRF in mediating the negative affective responses to stress and drug withdrawal (33).

The neuroanatomical entity termed the extended amygdala (34) may represent a common anatomical substrate that integrates brain arousal-stress systems with hedonic processing systems to produce the neuroadaptations associated with the development of addiction (see below). The extended amygdala is composed of the CeA, BNST, and a transition zone in the medial (shell) subregion of the nucleus accumbens. Each of these regions has cytoarchitectural and circuitry similarities (34). The extended amygdala receives numerous afferents from limbic structures, such as the basolateral amygdala and hippocampus, and sends efferents to the medial part of the ventral pallidum and a large projection to the lateral hypothalamus, thus further defining the specific brain areas that interface classical limbic (emotional) structures with the extrapyramidal motor system (35). CRF in the extended amygdala has long been hypothesized to play a key role not only in fear conditioning (36, 37) but also in the emotional component of pain processing (38).

### Dynorphin-κ opioid system

Dynorphins are opioid peptides that derive from the prodynorphin precursor and contain the leucine (leu)-enkephalin sequence at the *N*-terminal portion of the molecule and are the presumed endogenous ligands for the κ opioid receptor (39). Dynorphins are widely distributed in the central nervous system (40) and play a role in neuroendocrine regulation, pain regulation, motor activity, cardiovascular function, respiration, temperature regulation, feeding behavior, and stress responsivity (41). Dynorphins bind to all three opioid receptors but show a preference for κ receptors (39). Dynorphin-κ receptor system activation produces some actions that are similar to other opioids (analgesia) but others opposite to those of μ opioid receptors in the motivational domain. Dynorphins produce aversive dysphoric-like effects in animals and humans and have been hypothesized to mediate negative emotional states (42–45).

Dopamine receptor activation in the nucleus accumbens shell stimulates a cascade of events that ultimately lead to cyclic adenosine monophosphate response element-binding protein (CREB) phosphorylation and subsequent alterations in gene expression, notably the activation of the expression of prodynorphin mRNA. Subsequent activation of dynorphin systems has been hypothesized to feed back to decrease dopamine release in the mesolimbic dopamine system (46–50) and glutamate release in the nucleus accumbens (51, 52). Both of these changes may contribute to the dysphoric syndrome associated with cocaine dependence. *In vivo* microdialysis studies have also provided evidence that κ opioid receptors located in the prefrontal cortex (PFC) and ventral tegmental area also regulate the basal activity of mesocortical dopamine neurons (53, 54). In the extended amygdala, enhanced dynorphin action may also activate brain stress responses, such as CRF (55), or CRF in turn may activate dynorphin (56, 57).

## Dynamic Changes in Reward: Opponent Process

**Figure 3:**
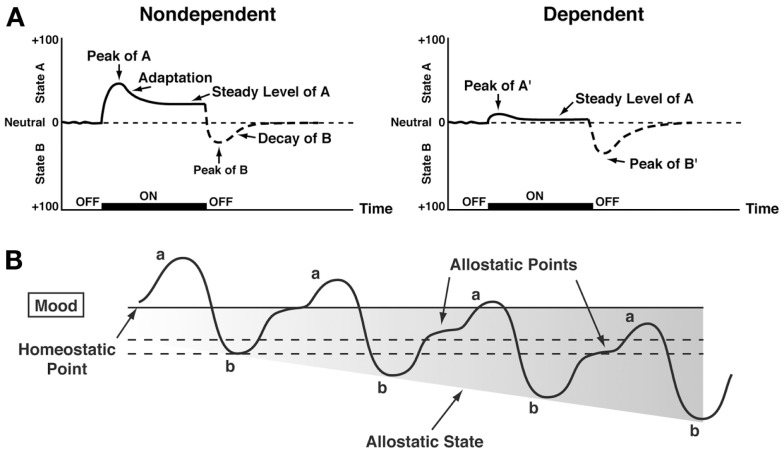
**(A)** The standard pattern of affective dynamics produced by (*left*) a relatively novel unconditioned stimulus (i.e., in a non-dependent state) and (*right*) a familiar, frequently repeated unconditioned stimulus (i.e., in a dependent state) [taken with permission from Ref. (184)]. **(B)** The changes in the affective stimulus (state) in an individual with repeated frequent drug use that may represent a transition to an allostatic state in the brain reward systems and, by extrapolation, a transition to addiction. Note that the apparent *b-process* never returns to the original homeostatic level before drug taking is reinitiated, thus creating a greater and greater allostatic state in the brain reward system. In other words, the counteradaptive opponent-process (*b-process*) does not balance the activational process (*a-process*) but in fact shows a residual hysteresis. While these changes are exaggerated and condensed over time in the present conceptualization, the hypothesis here is that even during post-detoxification, a period of “protracted abstinence,” the reward system is still bearing allostatic changes. In the non-dependent state, reward experiences are normal, and the brain stress systems are not greatly engaged. During the transition to the state known as addiction, the brain reward system is in a major underactivated state while the brain stress system is highly activated [taken with permission from Ref. (15)].

Changes in reinforcement were inextricably linked with hedonic, affective, or emotional states in addiction in the context of temporal dynamics by Solomon’s opponent-process theory of motivation. Solomon and Corbit (58) postulated that hedonic, affective, or emotional states, once initiated, are automatically modulated by the central nervous system through mechanisms that reduce the intensity of hedonic feelings. The *a-process* includes affective or hedonic habituation (or tolerance), and the *b-process* includes affective or hedonic withdrawal (abstinence). The *a-process* in drug use consists of positive hedonic responses, occurs shortly after the presentation of a stimulus, correlates closely with the intensity, quality, and duration of the reinforcer, and shows tolerance. In contrast, the *b-process* in drug use appears after the *a-process* has terminated, consists of negative hedonic responses, and is sluggish in onset, slow to build up to an asymptote, slow to decay, and gets larger with repeated exposure. The thesis we have elaborated is that there is a neurocircuitry change in specific neurochemical systems that account for the *b-process*. Such opponent processes are hypothesized to begin early in drug taking, reflecting not only deficits in brain reward system function but also the recruitment of brain stress systems. Furthermore, we hypothesize that the recruitment of brain stress systems forms one of the major sources of negative reinforcement in addiction. Finally, we have hypothesized that such changes result not in a return to homeostasis of reward/stress function but in allostasis of reward/stress function that continues to drive the addiction process (Figure 3).

Allostasis, originally conceptualized to explain persistent morbidity of arousal and autonomic function, can be defined as “stability through change.” Allostasis involves a feed-forward mechanism rather than the negative feedback mechanisms of homeostasis, with continuous reevaluation of need and continuous readjustment of all parameters toward new set points. An *allostatic state* has been defined as a state of chronic deviation of the regulatory system from its normal (homeostatic) operating level (15). *Allostatic load* was defined as the “long-term cost of allostasis that accumulates over time and reflects the accumulation of damage that can lead to pathological states” (59).

Opponent process-like negative emotional states have been characterized in humans by acute and protracted abstinence from all major drugs of abuse (60–62). Similar results have been observed in animal models with all major drugs of abuse using intracranial self-stimulation (ICSS) as a measure of hedonic tone. Withdrawal from chronic cocaine (63), amphetamine (64), opioids (65), cannabinoids (66), nicotine (67), and ethanol (68) leads to increases in reward threshold during acute abstinence, and some of these elevations in threshold can last for up to 1 week (69). These observations lend credence to the hypothesis that opponent processes in the hedonic domain have an identifiable neurobiological basis and provide an impetus for defining the mechanisms involved. Understanding the mechanisms that drive this increase in reward thresholds is key to understanding the mechanisms that drive negative reinforcement in addiction.

Such elevations in reward threshold begin rapidly and can be observed within a single session of self-administration (70), bearing a striking resemblance to human subjective reports of acute withdrawal. Dysphoria-like responses also accompany acute opioid and ethanol withdrawal (71, 72). Here, naloxone administration following single injections of morphine increased reward thresholds, measured by ICSS, and increased thresholds with repeated morphine and naloxone-induced withdrawal experience (71). Similar results were observed during repeated acute withdrawal from ethanol (72).

## Neuroadaptations Responsible for Opponent Process

One hypothesis is that drug addiction progresses from a source of positive reinforcement that may indeed involve a form of sensitization of incentive salience, as argued by Robinson and Berridge (9), to sensitization of opponent processes that set up a powerful negative reinforcement process. A further elaboration of this hypothesis is that there are both within- and between-system neuroadaptations to excessive activation of the reward system at the neurocircuitry level. Within-system neuroadaptations are defined as the process by which the primary cellular response element to the drug (circuit A) itself adapts to neutralize the drug’s effects. Persistence of the opposing effects after the drug disappears produces adaptation. A between-system neuroadaptation is a circuitry change, in which B circuits (i.e., the stress or anti-reward circuits) are activated by circuit A (i.e., the reward circuit). In the present treatise, within-system neuroadaptations can dynamically interact with a between-system neuroadaptation, in which circuit B (i.e., the anti-reward circuit) is activated either in parallel or in series to suppress the activity of circuit A (see below).

### Animal models of the transition to an addiction-like state as defined by escalation in drug self-administration with prolonged access

A progressive increase in the frequency and intensity of drug use is one of the major behavioral phenomena that characterize the development of addiction and has face validity with the criteria of the *Diagnostic and Statistical Manual of Mental Disorders*, 4th edition (DSM-IV): “The substance is often taken in larger amounts and over a longer period than was intended” (American Psychological Association, 1994). A framework with which to model the transition from drug use to drug addiction can be found in recent animal models of prolonged access to intravenous cocaine self-administration. Historically, animal models of cocaine self-administration involved the establishment of stable behavior from day to day to allow the reliable interpretation of data provided by within-subject designs aimed at exploring the neuropharmacological and neurobiological bases of the reinforcing effects of acute cocaine. Up until 1998, after the acquisition of self-administration, rats were typically allowed access to cocaine for 3 h or less per day to establish highly stable levels of intake and patterns of responding between daily sessions. This was a useful paradigm for exploring the neurobiological substrates for the acute reinforcing effects of drugs of abuse.

**Figure 4:**
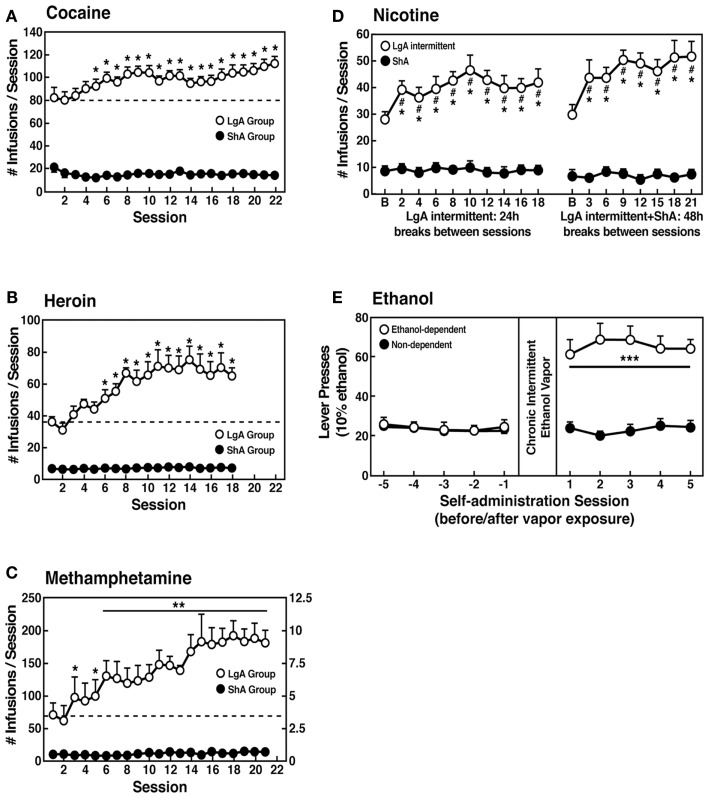
**(A)** Effect of drug availability on cocaine intake (mean ± SEM). In long-access (LgA) rats (*n* = 12) but not short-access (ShA) rats (*n* = 12), the mean total cocaine intake started to increase significantly from session 5 (*p* < 0.05; sessions 5–22 compared with session 1) and continued to increase thereafter (*p* < 0.05; session 5 compared with sessions 8–10, 12, 13, and 17–22) [taken with permission from Ref. (74)]. **(B)** Effect of drug availability on total intravenous heroin self-infusions (mean ± SEM). During the escalation phase, rats had access to heroin (40 μg per infusion) for 1 h (ShA rats, *n* = 5–6) or 11 h per session (LgA rats, *n* = 5–6). Regular 1 h (ShA rats) or 11 h (LgA rats) sessions of heroin self-administration were performed 6 days a week. The dotted line indicates the mean ± SEM number of heroin self-infusions in LgA rats during the first 11 h session. *p < 0.05, different from the first session (paired *t*-test) [taken with permission from Ref. (73)]. **(C)** Effect of extended access to intravenous methamphetamine on self-administration as a function of daily sessions in rats trained to self-administer 0.05 mg/kg/infusion of intravenous methamphetamine during the 6 h session. ShA, 1 h session (*n* = 6). LgA, 6 h session (0.05 mg/kg/infusion, *n* = 4). **p < 0.01, compared with day 1 [taken with permission from Ref. (75)]. **(D)** Nicotine intake (mean ± SEM) in rats that self-administered nicotine under a fixed-ratio (FR) 1 schedule in either 21 h (LgA) or 1 h (ShA) sessions. LgA rats increased their nicotine intake on an intermittent schedule with 24–48 h breaks between sessions, whereas LgA rats on a daily schedule did not. The left shows the total number of nicotine infusions per session when the intermittent schedule included 24 h breaks between sessions. The right shows the total number of nicotine infusions per session when the intermittent schedule included 48 h breaks between sessions. ^#^*p* < 0.05, compared with baseline; *p < 0.05, compared with daily self-administration group. *n* = 10 per group [taken with permission from Ref. (185)]. **(E)** Ethanol self-administration in ethanol-dependent and non-dependent animals. The induction of ethanol dependence and correlation of limited ethanol self-administration before and excessive drinking after dependence induction following chronic intermittent ethanol vapor exposure is shown. ***p < 0.001, significant group × test session interaction. With all drugs, escalation is defined as a significant increase in drug intake within-subjects in extended-access groups, with no significant changes within-subjects in limited-access groups [taken with permission from Ref. (186)].

However, in an effort to explore the possibility that differential access to drugs of abuse may have more face validity for the compulsive-like responding observed in addiction, animals have been allowed extended access to all major drugs of abuse (Figure 4). Increased intake was observed in the extended-access group for intravenous cocaine, methamphetamine, heroin, and nicotine and oral alcohol during extended access and dependence (73–79). For example, when animals were allowed access for 1 and 6 h to different doses of cocaine, after escalation, both the long-access (LgA) and short-access (ShA) animals titrated their cocaine intake, but LgA rats consistently self-administered almost twice as much cocaine at any dose tested, further suggesting an upward shift in the set point for cocaine reward in the escalated animals (80–82).

**Figure 5:**
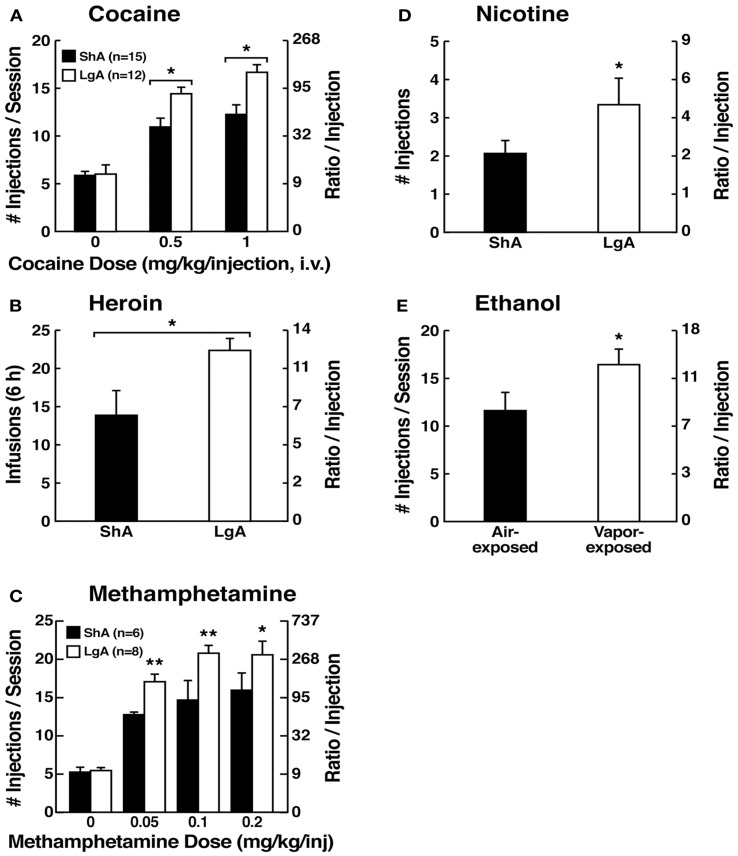
**(A)** Dose-response function of cocaine by rats responding under a progressive-ratio schedule. Test sessions under a progressive-ratio schedule ended when rats did not achieve reinforcement within 1 h. The data are expressed as the number of injections per session on the left axis and ratio per injection on the right axis. *p < 0.05, compared with ShA rats at each dose of cocaine [taken with permission from Ref. (84)]. **(B)** Responding for heroin under a progressive-ratio schedule of reinforcement in ShA and LgA rats. *p < 0.05, LgA significantly different from LgA [Modified with permission from Ref. (187)]. **(C)** Dose-response for methamphetamine under a progressive-ratio schedule. Test sessions under a progressive-ratio schedule ended when rats did not achieve reinforcement within 1 h. *p < 0.05, **p < 0.01, LgA significantly different from ShA [Modified from Ref. (188)]. **(D)** Breakpoints on a progressive-ratio schedule in long-access (LgA) rats that self-administered nicotine with 48 h abstinence between sessions. LgA rats on an intermittent schedule reached significantly higher breakpoints than LgA rats that self-administered nicotine daily. The data are expressed as mean ± SEM. *p < 0.05. *n* = 9 rats per group [taken with permission from Ref. (185)]. **(E)** Mean (±SEM) breakpoints for ethanol while in non-dependent and ethanol-dependent states. **p < 0.01, main effect of vapor exposure on ethanol self-administration [taken with permission from Ref. (85)].

Consistent with the hypothesis that extended access to drugs of abuse produces compulsive-like responding, in which animals will “continue to respond in the face of adverse consequences” (another DSM-IV criteria for Substance Dependence), animals with extended access that show escalation in self-administration also show increased responding on a progressive-ratio schedule of reinforcement [(83–85); Figure 5]. Changes in the reinforcing and incentive effects of drug intake that are consistent with the increases in progressive-ratio responding have been observed following extended access and include increased drug-induced reinstatement after extinction, a decreased latency to goal time in a runway model for drug reward, and responding in the face of punishment (86–92). Altogether, these results suggest that drug taking with extended-access changes the motivation to seek the drug. Some have argued that enhanced drug taking reflects a sensitization of reward (93), but studies of locomotor sensitization suggest that locomotor sensitization occurs independently of escalation (94–96). The increased brain reward thresholds and neuropharmacological studies outlined below argue for a reward deficit state that drives the increased drug taking during extended access.

### Animals escalate their intake of drugs with extended access, with a parallel increase in reward thresholds

**Figure 6:**
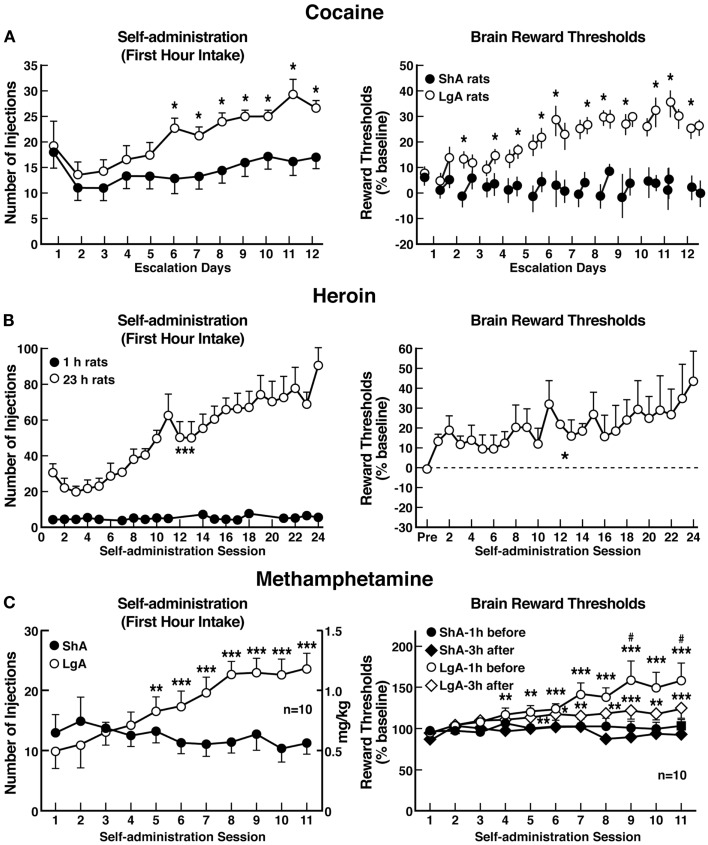
**(A)** Relationship between elevation in ICSS reward thresholds and cocaine intake escalation (*Left*). Percent change from baseline response latencies (3 h and 17–22 h after each self-administration session; first data point indicates 1 h before the first session) (*Right*). Percent change from baseline ICSS thresholds. *p < 0.05, compared with drug-naive and/or ShA rats (tests for simple main effects) [taken with permission from Ref. (97)]. **(B)** Unlimited daily access to heroin escalated heroin intake and decreased the excitability of brain reward systems (*Left*). Heroin intake (±SEM; 20 μg per infusion) in rats during limited (1 h) or unlimited (23 h) self-administration sessions. ***p < 0.001, main effect of access (1 or 23 h) (*Right*). Percent change from baseline ICSS thresholds (±SEM) in 23 h rats. Reward thresholds, assessed immediately after each daily 23 h self-administration session, became progressively more elevated as exposure to self-administered heroin increased across sessions. *p < 0.05, main effect of heroin on reward thresholds [taken with permission from Ref. (99)]. **(C)** Escalation in methamphetamine self-administration and ICSS in rats. Rats were daily allowed to receive ICSS in the lateral hypothalamus 1 h before and 3 h after intravenous methamphetamine self-administration with either 1 or 6 h access (*Left*). Methamphetamine self-administration during the first hour of each session (*Right*). ICSS measured 1 h before and 3 h after methamphetamine self-administration. *p < 0.05, **p < 0.01, ***p < 0.001, compared with session 1. ^#^*p* < 0.05, compared with LgA 3 h after [taken with permission from Ref. (98)].

The hypothesis that compulsive cocaine use is accompanied by a chronic perturbation in brain reward homeostasis has been tested in animal models of escalation in drug intake with prolonged access combined with measures of brain stimulation reward thresholds. Animals implanted with intravenous catheters and allowed differential access to intravenous self-administration of cocaine showed increases in cocaine self-administration from day to day in the LgA group (6 h; LgA) but not in the ShA group (1 h; ShA). The differential exposure to cocaine self-administration had dramatic effects on reward thresholds that progressively increased in LgA rats but not ShA or control rats across successive self-administration sessions (97). Elevations in baseline reward thresholds temporally preceded and were highly correlated with escalation in cocaine intake (Figure 6). Post-session elevations in reward thresholds failed to return to baseline levels before the onset of each subsequent self-administration session, thereby deviating more and more from control levels. The progressive elevation in reward thresholds was associated with a dramatic escalation in cocaine consumption that was observed previously (97). Similar results have been observed with extended access to methamphetamine (98) and heroin (99). Rats allowed 6 h access to methamphetamine or 23 h access to heroin also showed a time-dependent increase in reward thresholds that paralleled the increases in heroin intake (Figure 6). Similar results of parallel increases in brain reward thresholds with escalation of nicotine intake have been observed with extended access to nicotine (100).

## Brain Reward System Substrates for the Negative Reinforcement Associated with Addiction (Within-System Neuroadaptations)

**Figure 7:**
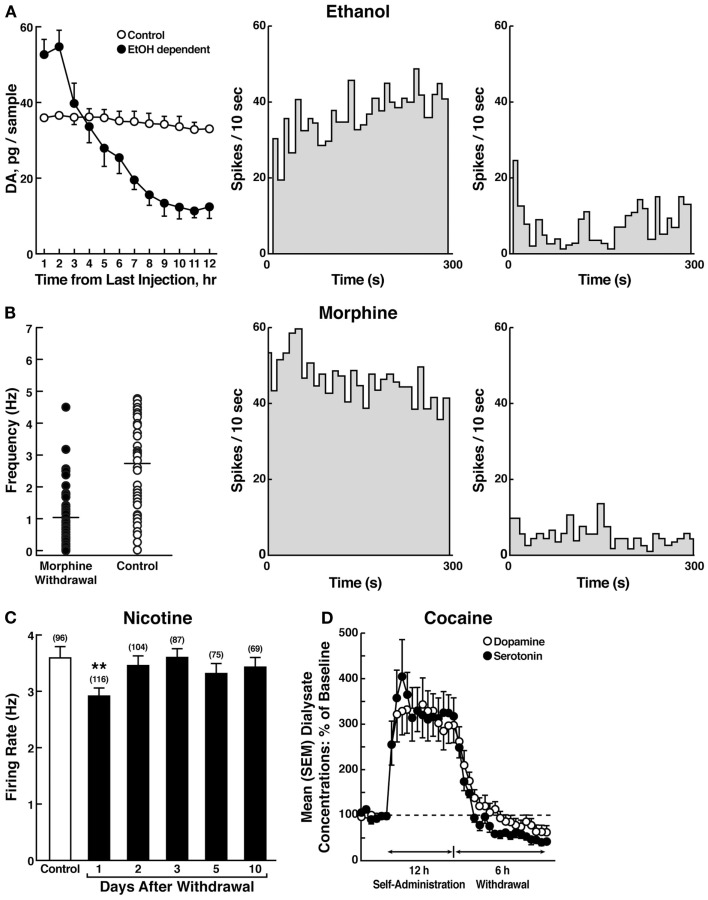
**(A)** The left panel shows the effect of ethanol withdrawal on absolute extracellular dopamine concentrations in the nucleus accumbens in ethanol-withdrawn rats. The middle and right panels show the spontaneous activity of antidromically identified ventral tegmental area-nucleus accumbens dopamine neurons in control (*middle*) and ethanol-withdrawn (*right*) rats [taken with permission from Ref. (102)]. **(B)** The left panel shows individual firing rates of antidromically identified ventral tegmental area-nucleus accumbens dopamine neurons recorded from morphine-withdrawn and control rats. Each circle represents the mean firing of at least a 5-min recording. Horizontal lines indicate the mean activity. The middle and right panels show the spontaneous activity of a selected number (4) or antidromically identified ventral tegmental area-nucleus accumbens dopamine neurons in control (*middle*) and morphine-withdrawn (*right*) rats. Each panel represents the neuronal activity of a single cell. Recordings in both cases were obtained 24 h after the last morphine and saline administration, respectively [taken with permission from Ref. (103)]. **(C)** Firing rates of dopamine cells in the ventral tegmental area following 1–10 days of withdrawal from chronic nicotine treatment (6 mg/kg/day for 12 days). The data are expressed as mean ± SEM. The number of dopamine cells recorded is given in parentheses. *p < 0.01, compared with control group [taken with permission from Ref. (189)]. **(D)** Profile of dialysate serotonin and dopamine concentrations during a 12-h extended-access cocaine self-administration session. The mean ± SEM presession baseline dialysate concentrations of serotonin and dopamine were 0.98 ± 0.1 nM and 5.3 ± 0.5 nM, respectively (*n* = 7) [taken with permission from Ref. (104)].

The *withdrawal/negative affect* stage can be defined as the presence of motivational signs of withdrawal in humans, including chronic irritability, physical pain, emotional pain [i.e., hyperkatifeia; (101)], malaise, dysphoria, alexithymia, and loss of motivation for natural rewards. It is characterized in animals by increases in reward thresholds during withdrawal from all major drugs of abuse. More compelling, as noted above, in animal models of the transition to addiction, similar changes in brain reward thresholds occur that temporally precede and are highly correlated with escalation in drug intake (97–99). Such acute withdrawal is associated with decreased activity of the mesocorticolimbic dopamine system, reflected by electrophysiological recordings and *in vivo* microdialysis [(102–104); Figure 7].

Human imaging studies of individuals with addiction during withdrawal or protracted abstinence have generated results that are consistent with animal studies. There are decreases in dopamine D_2_ receptors (hypothesized to reflect hypodopaminergic functioning), hyporesponsiveness to dopamine challenge (105), and hypoactivity of the orbitofrontal-infralimbic cortex system (105). These are hypothesized to be within-system neuroadaptations that may reflect presynaptic release or postsynaptic receptor plasticity.

In the context of chronic alcohol administration, multiple molecular mechanisms have been hypothesized to counteract the acute effects of ethanol that could be considered within-system neuroadaptations. For example, chronic ethanol decreases γ-aminobutyric acid (GABA) receptor function, possibly through downregulation of the α_1_ subunit (106, 107). Chronic ethanol also decreases the acute inhibition of adenosine reuptake [i.e., tolerance develops to the inhibition of adenosine by ethanol; (108)]. Perhaps more relevant to the present treatise, whereas acute ethanol activates adenylate cyclase, withdrawal from chronic ethanol decreases CREB phosphorylation in the amygdala and is linked to decreases in the function of NPY and anxiety-like responses observed during acute ethanol withdrawal (109, 110).

## Brain Stress System Substrates for the Negative Reinforcement Associated with Addiction (Between-System Neuroadaptations)

Brain neurochemical systems involved in arousal-stress modulation have been hypothesized to be engaged within the neurocircuitry of the brain stress systems in an attempt to overcome the chronic presence of the perturbing drug and restore normal function despite the presence of drug (18). Both the hypothalamic-pituitary-adrenal (HPA) axis and extrahypothalamic brain stress system mediated by CRF are dysregulated by chronic administration of all major drugs with dependence or abuse potential, with a common response of elevated adrenocorticotropic hormone, corticosterone, and amygdala CRF during acute withdrawal (24, 69, 111–116). Indeed, activation of the HPA response may be an early dysregulation associated with excessive drug taking that ultimately “sensitizes” the extrahypothalamic CRF systems (33, 92).

As noted above, the excessive release of dopamine and opioid peptides produces subsequent activation of dynorphin systems, which has been hypothesized to feed back to decrease dopamine release and also contribute to the dysphoric syndrome associated with cocaine dependence (48). Dynorphins produce aversive dysphoric-like effects in animals and humans and have been hypothesized to mediate negative emotional states (42–45).

A common response to acute withdrawal and protracted abstinence from all major drugs of abuse is the manifestation of anxiety-like responses that are reversed by CRF antagonists. Withdrawal from repeated administration of cocaine produces an anxiogenic-like response in the elevated plus maze and defensive burying test, both of which are reversed by administration of CRF receptor antagonists (117, 118). Opioid dependence also produces irritability-like effects that are reversed by CRF receptor antagonists (119, 120). Ethanol withdrawal produces anxiety-like behavior that is reversed by intracerebroventricular administration of CRF_1_/CRF_2_ peptidergic antagonists (121) and small-molecule CRF_1_ antagonists (122–124) and intracerebral administration of a peptidergic CRF_1_/CRF_2_ antagonist into the amygdala (125). Thus, some effects of CRF antagonists have been localized to the CeA (125). Precipitated withdrawal from nicotine produces anxiety-like responses that are also reversed by CRF antagonists (77, 126). CRF antagonists injected intracerebroventricularly or systemically also block the potentiated anxiety-like responses to stressors observed during protracted abstinence from chronic ethanol (127–131).

Another measure of negative emotional states during drug withdrawal in animals is conditioned place aversion, in which animals avoid an environment previously paired with an aversive state. Such place aversions, when used to measure the aversive stimulus effects of withdrawal, have been observed largely in the context of opioids (132, 133). Systemic administration of a CRF_1_ receptor antagonist and direct intracerebral administration of a peptide CRF_1_/CRF_2_ antagonist also decreased opioid withdrawal-induced place aversions (134–136). These effects have been hypothesized to be mediated by actions in the extended amygdala. The selective CRF_1_ antagonist antalarmin blocked the place aversion produced by naloxone in morphine-dependent rats (134), and a CRF peptide antagonist injected into the CeA also reversed the place aversion produced by methylnaloxonium injected into the CeA (135). CRF_1_ knockout mice failed to show conditioned place aversion to opioid withdrawal and failed to show an opioid-induced increase in dynorphin mRNA in the nucleus accumbens (136).

A compelling test of the hypothesis that CRF-induced increases in anxiety-like responses during drug withdrawal has motivational significance in contributing to negative emotional states is the observation that CRF antagonists can reverse the elevation in reward thresholds produced by drug withdrawal. Nicotine and alcohol withdrawal-induced elevations in reward thresholds were reversed by a CRF antagonist (137, 138). These effects have been localized to both the CeA and nucleus accumbens shell (139).

Enhanced dynorphin action is hypothesized to mediate the depression-like, aversive responses to stress, and dysphoric-like responses during withdrawal from drugs of abuse (49, 56, 57, 140–145). For example, pretreatment with a κ-opioid receptor antagonist blocked stress-induced analgesia and stress-induced immobility (57), decreased anxiety-like behavior in the elevated plus maze and open field, decreased conditioned fear in fear-potentiated startle (145), and blocked depressive-like behavior induced by cocaine withdrawal (140).

## Brain Stress Substrates that Mediate Drug Taking with Extended Access

### Corticotropin-releasing factor, compulsive-like drug seeking, and the extended amygdala

**Figure 8:**
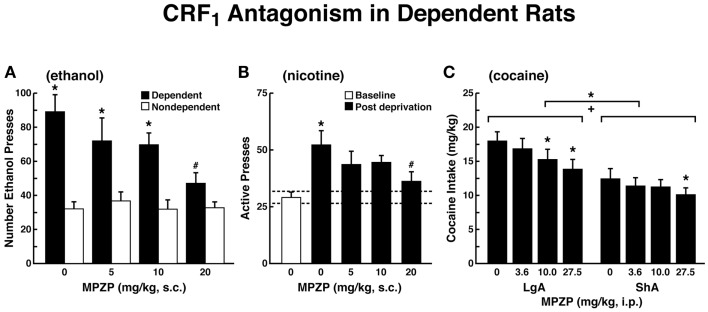
**Effects of CRF_1_ antagonist on compulsive-like responding for drugs of abuse in rats with extended access to drug (A)**. The effect of the CRF_1_ receptor antagonist MPZP on operant self-administration of alcohol in dependent and non-dependent rats. Testing was conducted when dependent animals were in acute withdrawal (6–8 h after removal from vapors). Dependent rats self-administered significantly more than non-dependent animals, and MPZP dose-dependently reduced alcohol self-administration only in dependent animals. The data are expressed as mean + SEM lever presses for alcohol [taken with permission from Ref. (190)]. **(B)** Abstinence-induced escalation of nicotine intake is blocked by a CRF_1_ receptor antagonist. Effect of MPZP (s.c., −1 h) on nicotine self-administration during the active period in rats given extended access to nicotine. *p < 0.05, compared with baseline; ^#^*p* < 0.05, compared with after-abstinence vehicle treatment; *n* = 8). The data are expressed as mean + SEM lever presses for nicotine [taken with permission from Ref. 77)]. **(C)** MPZP reduces cocaine intake in ShA and LgA rats. The data are expressed as mean + SEM cocaine intake (mg/kg). *p < 0.05, **p < 0.01, compared with vehicle [taken with permission from Ref. (146)].

The ability of CRF antagonists to block the anxiogenic-like and aversive-like motivational effects of drug withdrawal predicted motivational effects of CRF antagonists in animal models of extended access to drugs. CRF antagonists selectively blocked the increased self-administration of drugs associated with extended access to intravenous self-administration of cocaine (146), nicotine (77), and heroin [(147); Figure 8]. For example, systemic administration of a CRF_1_ antagonist blocked the increased self-administration of nicotine associated with withdrawal in extended-access (23 h) animals (77).

Corticotropin-releasing factor antagonists also blocked the increased self-administration of ethanol in dependent rats [(124); Figure 8]. For example, exposure to repeated cycles of chronic ethanol vapor produced substantial increases in ethanol intake in rats during both acute withdrawal and protracted abstinence [2 weeks post-acute withdrawal; (76, 148)]. Intracerebroventricular administration of a CRF_1_/CRF_2_ antagonist blocked the dependence-induced increase in ethanol self-administration during both acute withdrawal and protracted abstinence (149). Systemic injections of small-molecule CRF_1_ antagonists also blocked the increased ethanol intake associated with acute withdrawal (124) and protracted abstinence (150). When administered directly into the CeA, a CRF_1_/CRF_2_ antagonist blocked ethanol self-administration in ethanol-dependent rats (151). These effects appear to be mediated by the actions of CRF on GABAergic interneurons within the CeA, and a CRF antagonist administered chronically during the development of dependence blocked the development of compulsive-like responding for ethanol (116). Altogether, these results suggest that CRF in the basal forebrain may also play an important role in the development of the aversive motivational effects that drive the increased drug-seeking associated with cocaine, heroin, nicotine, and alcohol dependence.

### Dynorphin, compulsive-like drug seeking, and the extended amygdala

Recent evidence suggests that the dynorphin-κ opioid system also mediates compulsive-like drug responding (methamphetamine, heroin, and alcohol) with extended access and dependence. Evidence from our laboratory has shown a small-molecule κ antagonist selectively blocked responding on a progressive-ratio schedule for cocaine in rats with extended access (152). Even more compelling is that excessive drug self-administration can also be blocked by κ antagonists (152–155) and may be mediated by the shell of the nucleus accumbens (156). However, the neurobiological circuits involved in mediating the effects of activation of the dynorphin-κ opioid system on the escalation of methamphetamine intake with extended access, remain unknown.

### NPY, compulsive drug seeking, and the extended amgydala

Neuropeptide Y is a neuropeptide with dramatic anxiolytic-like properties localized to multiple brain regions but heavily innervating the amygdala. It is hypothesized to have effects opposite to CRF in the negative motivational state of withdrawal from drugs of abuse and as such increases in NPY function may act in opposition to the actions of increases in CRF (157). Significant evidence suggests that activation of NPY in the CeA can block the motivational aspects of dependence associated with chronic ethanol administration. NPY administered intracerebroventricularly blocked the increased drug intake associated with ethanol dependence (158, 159). NPY also decreased excessive alcohol intake in alcohol-preferring rats (160). Injection of NPY directly into the CeA (161) and viral vector-enhanced expression of NPY in the CeA also blocked the increased drug intake associated with ethanol dependence (162). At the cellular level, NPY, like CRF_1_ antagonists, blocks the increase in GABA release in the CeA produced by ethanol and also when administered chronically blocks the transition to excessive drinking with the development of dependence (163). The role of NPY in the actions of other drugs of abuse is limited, particularly with regard to dependence and compulsive drug seeking. NPY_5_ receptor knockout mice have a blunted response to the rewarding effects of cocaine (164, 165), and NPY knockout mice show hypersensitivity to cocaine self-administration (166). NPY itself injected intracerebroventricularly facilitated heroin and cocaine self-administration and induced reinstatement of heroin seeking in limited-access rats (167, 168). An NPY Y_2_ antagonist, possibly acting presynaptically to release NPY, blocked social anxiety associated with nicotine withdrawal (169), and NPY injected intracerebroventricularly blocked the somatic signs but not reward deficits associated with nicotine withdrawal (170). However, the role of NPY in compulsive drug seeking with extended-access remains to be studied. The hypothesis here would be that NPY is a buffer or homeostatic response to between-system neuroadaptations that can return the brain emotional systems to homeostasis (157, 171).

#### Corticotropin-releasing factor, stress, and the frontal cortex

Converging lines of evidence suggest that impairment of medial PFC (mPFC) cognitive function and overactivation of the CeA may be linked to the development of compulsive-like responding for drugs of abuse during extended access (172–174). Extended access to cocaine self-administration induced an escalated pattern of cocaine intake associated with an impairment of working memory and decrease in the density of dorsomedial PFC (dmPFC) neurons that lasted for months after cocaine cessation (172). Whereas LgA and ShA rats exhibited a high percentage of correct responses in the delayed non-matching-to-sample task under low cognitive demand (delay < 10 s), increasing the working memory load (i.e., close to the capacity limit of working memory) by increasing the delay from 10 to 70 and 130 s revealed a robust working memory deficit in LgA rats. Furthermore, the magnitude of escalation of cocaine intake was negatively correlated with working memory performance in ShA and LgA rats with the 70- and 130-s delays but not with the 10-s delay or with baseline performance during training, demonstrating that the relationship between the escalation of cocaine intake and behavioral performance in this task was restricted to working memory performance under high cognitive demand. The density of neurons and oligodendrocytes in the dmPFC was positively correlated with working memory performance. A lower density of neurons or oligodendrocytes in the dmPFC was associated with more severe working memory impairment. Working memory was also correlated with the density of oligodendrocytes in the orbitofrontal cortex (OFC), suggesting that OFC alterations after escalated drug intake may play a role in working memory deficits. However, no correlation was found between working memory performance and neuronal density in the OFC, suggesting that OFC neurons may be less vulnerable to the deleterious effects of chronic cocaine exposure than dmPFC neurons. Thus, PFC dysfunction may exacerbate the loss of control associated with compulsive drug use and facilitate the progression to drug addiction.

Similar results have been observed in an animal model of binge alcohol consumption, even before the development of dependence. Using an animal model of escalation of alcohol intake with chronic intermittent access to alcohol, in which rats are given continuous (24 h per day, 7 days per week) or intermittent (3 days per week) access to alcohol (20% v/v) using a two-bottle choice paradigm, FBJ murine osteosarcoma viral oncogene homolog (Fos) expression in the mPFC, CeA, hippocampus, and nucleus accumbens were measured and correlated with working memory and anxiety-like behavior (175). Abstinence from alcohol in rats with a history of escalation of alcohol intake specifically recruited GABA and CRF neurons in the mPFC and produced working memory impairments associated with excessive alcohol drinking during acute (24–72 h) but not protracted (16–68 days) abstinence. The abstinence from alcohol was associated with a functional disconnection of the mPFC and CeA but not mPFC or nucleus accumbens. These results show that recruitment of a subset of GABA and CRF neurons in the mPFC during withdrawal and disconnection of the PFC CeA pathway may be critical for impaired executive control over motivated behavior, suggesting that dysregulation of mPFC interneurons may be an early index of neuroadaptation in alcohol dependence.

## Brain Stress Systems in Addiction: An Allostatic View

More importantly for the present thesis, as dependence and withdrawal develop, brain anti-reward systems, such as CRF and dynorphin, are recruited in the extended amygdala. We hypothesize that this brain stress neurotransmitter that is known to be activated during the development of excessive drug taking comprises a between-system opponent process, and this activation is manifest when the drug in removed, producing anxiety, hyperkatifeia, and irritability symptoms associated with acute and protracted abstinence. Notably, however, there is evidence of CRF immunoreactivity in the ventral tegmental area, and a CRF_1_ receptor antagonist injected directly into the ventral tegmental area blocked the social stress-induced escalation of cocaine self-administration (176). Altogether, these observations suggest between-system/within-system neuroadaptations that were originally hypothesized for dynorphin by Carlezon and Nestler (177), in which activation of CREB by excessive dopamine and opioid peptide receptor activation in the nucleus accumbens triggers the induction of dynorphin to feed back to suppress dopamine release. Thus, we hypothesize that anti-reward circuits are recruited as between-system neuroadaptations (178) during the development of addiction and produce aversive or stress-like states (179–181) via two mechanisms: direct activation of stress-like, fear-like states in the extended amygdala (CRF) and indirect activation of a depression-like state by suppressing dopamine (dynorphin).

A critical problem in drug addiction is chronic relapse, in which addicted individuals return to compulsive drug taking long after acute withdrawal. This corresponds to the *preoccupation/anticipation* stage of the addiction cycle outlined above. Koob and Le Moal also hypothesized that the dysregulations that comprise the “dark side” of drug addiction persist during protracted abstinence to set the tone for vulnerability to “craving” by activating drug-, cue-, and stress-induced reinstatement neurocircuits that are now driven by a reorganized and possibly hypofunctioning prefrontal system. The hypothesized allostatic, dysregulated reward, and sensitized stress state produces the motivational symptoms of acute withdrawal and protracted abstinence and provides the basis by which drug priming, drug cues, and acute stressors acquire even more power to elicit drug-seeking behavior (92). Thus, the combination of decreases in reward system function and recruitment of anti-reward systems provides a powerful source of negative reinforcement that contributes to compulsive drug-seeking behavior and addiction. A compelling argument can be made that the neuroplasticity that charges the CRF stress system may indeed begin much earlier that previously thought via stress actions in the PFC.

**Figure 9:**
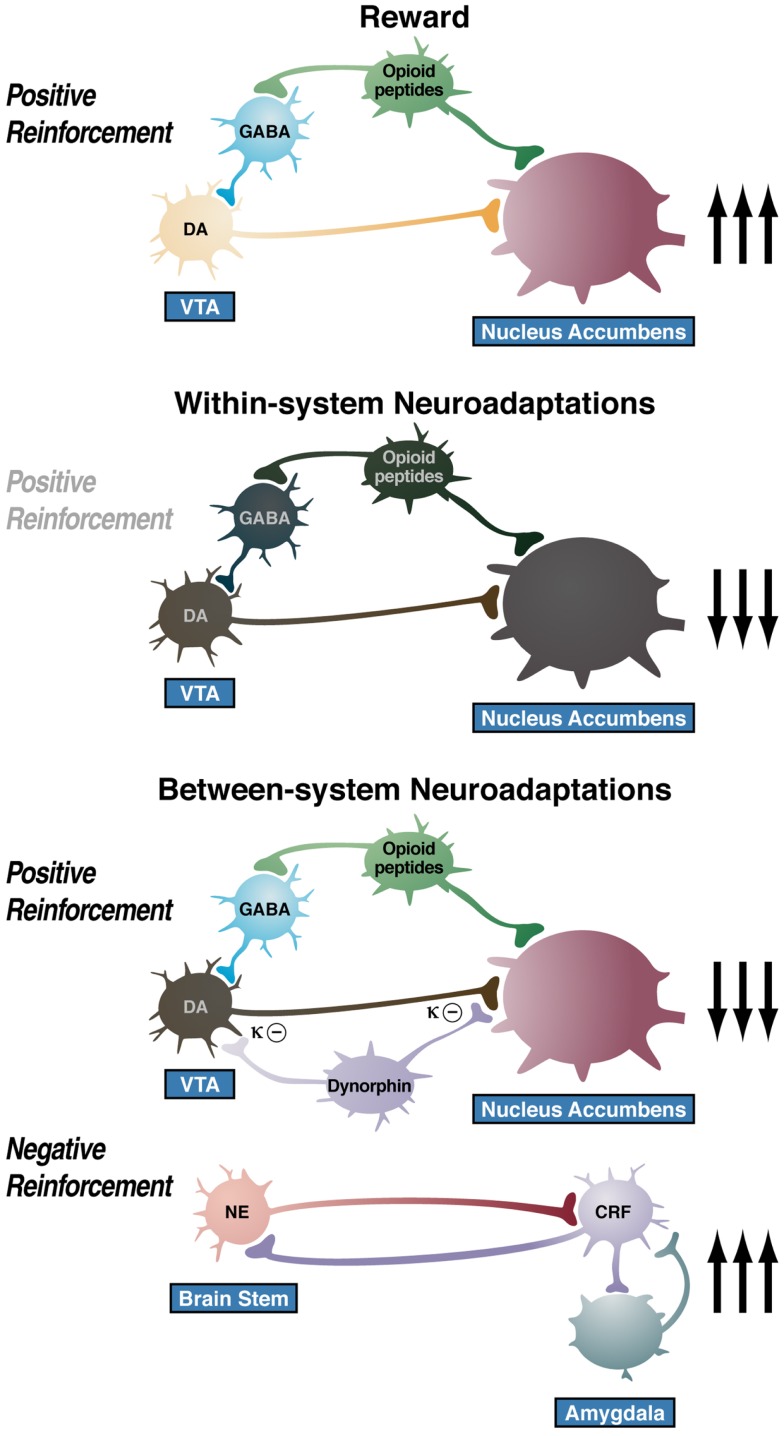
**Diagram of the hypothetical “within-system” and “between-system” changes that lead to the “darkness within**.” (Top) Circuitry for drug reward with major contributions from mesolimbic dopamine and opioid peptides that converge on the nucleus accumbens. During the *binge/intoxication* stage of the addiction cycle, the reward circuitry is excessively engaged, Middle. Such excessive activation of the reward system triggers “within-system” neurobiological adaptations during the *withdrawal/negative affect* stage, including activation of cyclic adenosine monophosphate (cAMP) and cAMP response element-binding protein (CREB), downregulation of dopamine D_2_ receptors, and decreased firing of ventral tegmental area (VTA) dopaminergic neurons, Bottom. As dependence progresses and the *withdrawal/negative affect* stage is repeated, two major “between-system” neuroadaptations occur. One is activation of dynorphin feedback that further decreases dopaminergic activity. The other is recruitment of extrahypothalamic norepinephrine (NE)-corticotropin-releasing factor (CRF) systems in the extended amygdala. Facilitation of the brain stress system in the prefrontal cortex is hypothesized to exacerbate the between-system neuroadaptations while contributing to the persistence of the dark side into the *preoccupation/anticipation* stage of the addiction cycle [taken with permission from Ref. (191)].

The overall conceptual theme argued here is that drug addiction represents an excessive and prolonged engagement of homeostatic brain regulatory mechanisms that regulate the response of the body to rewards and stressors. The dysregulation of the incentive salience systems may begin with the first administration of drug (182), and the dysregulation of the stress axis may begin with the binge and subsequent acute withdrawal, triggering a cascade of changes, from activation of the HPA axis to activation of CRF in the PFC to activation of CRF in the extended amygdala to activation of dynorphin in the ventral striatum (Figure 9). This cascade of overactivation of the stress axis represents more than simply a transient homeostatic dysregulation; it also represents the dynamic homeostatic dysregulation termed *allostasis*.

Repeated challenges, such as with drugs of abuse, lead to attempts of the brain stress systems at the molecular, cellular, and neurocircuitry levels to maintain stability but at a cost. For the drug addiction framework elaborated here, the residual decrease in the brain reward systems and activation of the brain stress systems to produce the consequent negative emotional state is termed an *allostatic state* (15). This state represents a combination of recruitment of anti-reward systems and consequent chronic decreased function of reward circuits, both of which lead to the compulsive drug seeking and loss of control over intake. How these systems are modulated by other known brain emotional systems localized to the basal forebrain, where the ventral striatum and extended amygdala project to convey emotional valence, how frontal cortex dysregulations in the cognitive domain are linked to impairments in executive function to contribute to the dysregulation of the extended amygdala, and how individuals differ at the molecular-genetic level of analysis to convey loading on these circuits remain challenges for future research.

## Conflict of Interest Statement

The authors declare that the research was conducted in the absence of any commercial or financial relationships that could be construed as a potential conflict of interest.
